# Flightless birds are not neuroanatomical analogs of non-avian dinosaurs

**DOI:** 10.1186/s12862-018-1312-0

**Published:** 2018-12-13

**Authors:** Maria Eugenia Leone Gold, Akinobu Watanabe

**Affiliations:** 1 Biology Department, Suffolk University, Boston, MA 02108 USA; 2 Department of Anatomical Sciences, Stony Brook University, Stony Brook, NY 11779 USA; 3 Division of Paleontology, American Museum of Natural History, New York, NY 10024 USA; 4 Department of Anatomy, New York Institute of Technology College of Osteopathic Medicine, Old Westbury, NY 11568 USA; 5 Life Sciences Department Vertebrates Division, Natural History Museum, London, SW7 5BD UK

**Keywords:** 3-D geometric morphometrics, Endocasts, Neuroanatomy, Theropoda, Aves, Powered flight, Locomotion

## Abstract

**Background:**

In comparative neurobiology, major transitions in behavior are thought to be associated with proportional size changes in brain regions. Bird-line theropod dinosaurs underwent a drastic locomotory shift from terrestrial to volant forms, accompanied by a suite of well-documented postcranial adaptations. To elucidate the potential impact of this locomotor shift on neuroanatomy, we first tested for a correlation between loss of flight in extant birds and whether the brain morphology of these birds resembles that of their flightless, non-avian dinosaurian ancestors. We constructed virtual endocasts of the braincase for 80 individuals of non-avian and avian theropods, including 25 flying and 19 flightless species of crown group birds. The endocasts were analyzed using a three-dimensional (3-D) geometric morphometric approach to assess changes in brain shape along the dinosaur-bird transition and secondary losses of flight in crown-group birds (Aves).

**Results:**

While non-avian dinosaurs and crown-group birds are clearly distinct in endocranial shape, volant and flightless birds overlap considerably in brain morphology. Phylogenetically informed analyses show that locomotory mode does not significantly account for neuroanatomical variation in crown-group birds. Linear discriminant analysis (LDA) also indicates poor predictive power of neuroanatomical shape for inferring locomotory mode. Given current sampling, *Archaeopteryx*, typically considered the oldest known bird, is inferred to be terrestrial based on its endocranial morphology.

**Conclusion:**

The results demonstrate that loss of flight does not correlate with an appreciable amount of neuroanatomical changes across Aves, but rather is partially constrained due to phylogenetic inertia, evident from sister taxa having similarly shaped endocasts. Although the present study does not explicitly test whether endocranial changes along the dinosaur-bird transition are due to the acquisition of powered flight, the prominent relative expansion of the cerebrum, in areas associated with flight-related cognitive capacity, suggests that the acquisition of flight may have been an important initial driver of brain shape evolution in theropods.

**Electronic supplementary material:**

The online version of this article (10.1186/s12862-018-1312-0) contains supplementary material, which is available to authorized users.

## Background

Major behavioral transitions often correlate with neuroanatomical changes because novel sensory inputs and motor control pathways can form new or more robust connections, increasing the volume and density of associated regions of the brain [1–5]. One such evolutionary transition, from non-volancy to powered flight, has been acquired independently by three different vertebrate groups—pterosaurs, bats, and birds [6]. Among these clades, birds provide arguably the best system within which to study the evolution of flight and associated morphological adaptations because they have a long stem lineage that is represented by a rich fossil record [7, 8]. This record has elucidated the origins of many of the postcranial ‘adaptations’ traditionally considered directly related to flight and has shown that these characters, such as the furcula and hollow long bones, arose not at the origin of this locomotory mode—near the appearance of *Archaeopteryx lithographica*—but earlier among non-avian theropods [9–11].

The early history of neuroanatomical adaptions related to this transition, however, is more obscure. Previous studies of avian neuroanatomical evolution indicated that the avian brain is characterized by an enlarged cerebrum and dorsoventrally flexed lateral profile. These studies divided endocasts into distinct volumes based on major brain regions (e.g. cerebrum, cerebellum, optic lobe). The fossil record shows that these ‘avian-like’ cerebral volumes evolved at least as far back as the origin of Maniraptora, ~ 160 Mya [11, 12]. This indicates that the size of the brain follows the more general trend of other ‘avian’ characteristics, evolving well before the origin of powered flight. Purely volumetric analyses, however, are limited in their capacity to characterize morphology. Neural pathways important in creating and regulating locomotor behavior often are distributed differentially among multiple regions of the brain [5], thus understanding the regional shape changes has the potential to inform the evolutionary tempo and mode of fight capacity along the theropod lineage.

Here, we use the loss of flight in crown group birds as a potential reverse analogue to the acquisition of flight in theropods. To determine if there is a link between neuroanatomy and loss of flight, we examined the endocasts of multiple independent pairs of flightless birds and their closest volant relatives to test for modifications in brain shape. We then compared the endocasts to the plesiomorphic condition in flightless, non-avian theropod dinosaurs. We used high-resolution X-ray computed tomographic (CT) imaging to construct three-dimensional (3-D) digital endocasts of the cranial cavity of a broad sample of modern flying and flightless members of Aves (sensu [13]) and extinct non-avian theropods. Shape data from these specimens were collected using a high-dimensional geometric morphometric (GM) approach [14–16] and they were subsequently subjected to a suite of multivariate analyses [17, 18]. With this approach, we evaluated whether (1) the loss of flight incurs predictable changes to neuroanatomical shape, such as a reduction in cerebral areas known to function in flight [5]; and (2) the endocast of modern flightless birds returns to a shape more similar to that of non-avian theropods than to their flying relatives.

## Methods

### CT imaging and digital endocast reconstruction

Taxonomic sampling included the skulls of 80 specimens representing 51 species: 25 flying and 19 flightless avians, and 7 non-avian dinosaurs: *Alioramus altai* [19] (IGM 100/1844), *Khaan mckennai* [20] (IGM 100/973), *Citipati osmolskae* [20] (IGM 100/978), *Incisivosaurus gauthieri* [21] (IVPP V 13326), *Zanabazar junior* [22] (IGM 100/1), an unnamed troodontid (IGM 100/1126 [13]) and *Archaeopteryx lithographica* [11] (NMNH PV OR 37001). We CT scanned each specimen using a General Electric v|tome Phoenix Computed Tomography (CT) scanner (General Electric, Heidelberg, Germany) at the Microscopy and Imaging Facility at the AMNH or the high-resolution source at the University of Texas at Austin High-Resolution X-ray Computed Tomography Facility (Additional file 1: Tables S1 and S2). *Raphus cucullatus* (NHMUK PV A9040) [23] was scanned at the NHMUK Imaging Facility. Scan data were imported into VGStudio MAX v2.2 (Volume Graphics GmbH, Heidelberg, Germany) to construct a 3-D model of the endocranial cavity (endocast) following the protocol set forth by Balanoff et al. [24]. Because the brain fills nearly the entire cranial cavity in birds [25, 26], its morphology is accurately reflected by use of an endocast [24, 27]. The 3-D models were smoothed in VGStudio MAX and exported as PLY files for the software Landmark Editor v3.6 to virtually place landmarks [28].

### Time-calibrated phylogenetic tree

A time-calibrated tree was used to perform comparative phylogenetic analyses on endocranial shape. For extant birds, we constructed a topology using the data provided online by Jetz et al. [29] (birdtree.org), pruned to include only species sampled in the current study. The Jetz et al. [29] trees were chosen for their dense species sampling. Our dataset has, in some cases, multiple species that are very closely related and the increased tip sampling of that tree allowed us to retain all of our species.

**Fig. 1:**
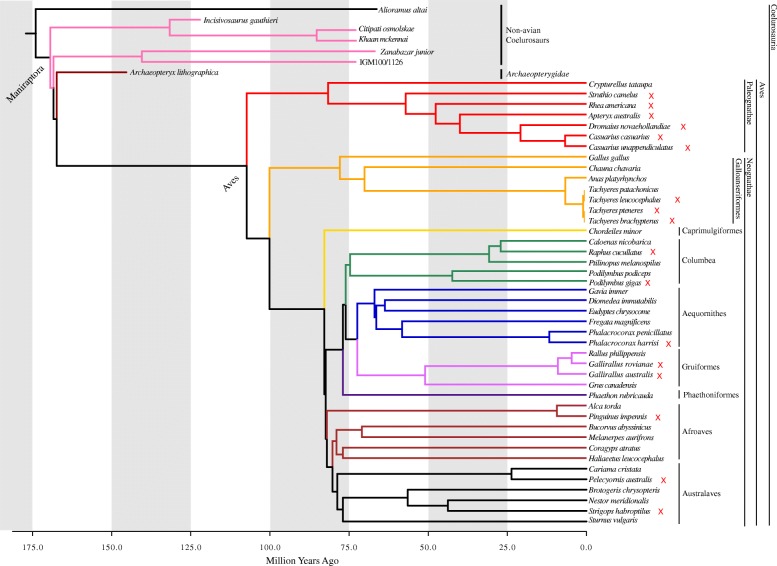
Time-calibrated phylogeny of taxa sampled in the study. Maximum clade credibility tree based on Jetz et al. [26] with a Hackett et al. [28] backbone. Non-avian dinosaurs and *Archaeopteryx* were incorporated into the phylogeny based on fossil occurrence data from Paleobiology Database (paleobiodb.org). Colors correspond to major clades and are consistent across figures. Red crosses next to taxonomic names indicate a flightless taxon

TreeAnnotator v1.8.1 [30] was used to construct a maximum clade credibility tree from 1000 posterior trees based on the Hackett et al. [31] tree backbone [29]. Because some of the sampled taxa are not included in the Jetz et al. [29] dataset, we replaced these tip labels with the closest relatives to each of these species in our analysis. Thus, *Alle alle, Chunga burmeisteri,* and *Goura cristata*, and *Rollandia rolland* were replaced by *Pinguinus impennis, Pelecyornis australis*, *Raphus cucullatus*, and *Podilymbus gigas*, respectively. We considered the above listed extinct crown group avians to exist in the Recent for the phylogeny (i.e., 0 Ma) in the context of deep time. The dataset included *Struthio molybdophanes* and *S. camelus*, however, only the latter was present in the Jetz et al. [29] dataset. Both species were included for the non-phylogenetically informed analyses (e.g. PCA and LDA), but only *S. camelus* was used for the construction of the time-calibrated tree and phylogenetically informed analyses. This tree was used for the crown-group Aves dataset (Fig. 1). For the Coelurosaur dataset, we used Mesquite v3.02 [32] to incorporate non-avian dinosaurs into the time-calibrated avian phylogeny based on the phylogenetic relationships proposed by Brusatte et al. [8]. Branch lengths were calculated for non-avian coelurosaurs based on oldest fossil ages from the Paleobiology Database (http://www.paleobiodb.org) in sister subclades for each internal node (e.g., the species range for *Incisivosaurus* is 122.46–125.45 Ma and for Oviraptorosauria is 130.0 Ma, based on the first fossil occurrence of *Caudipteryx zoui*; subtracting the first occurrence age from the latter gives 4.55 Myr, which is the branch length for *Incisivosaurus*). This combined extant and fossil tree was used for the Coelurosaur dataset (Fig. 1).

### Landmark data

**Fig. 2:**
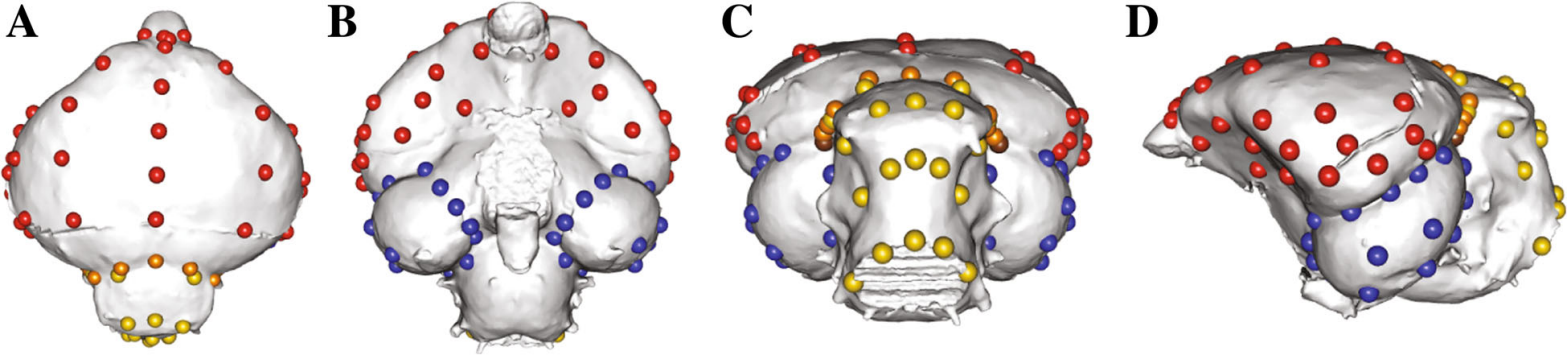
Landmark scheme on the endocast of *Crypturellus tataupa* (AMNH 604) in **a**) dorsal, **b**) ventral, **c**) caudal, and **d**) left lateral views. Cerebrum (red), cerebellum (yellow), optic lobes (blue), landmarks on the division between cerebrum and cerebellum (orange), triple point between optic lobe, cerebellum, and cerebrum (brown)

To collect landmark data, each 3-D endocranial reconstruction was imported into Landmark Editor [28] for digital placement of 3-D Cartesian coordinate points. The relatively featureless surface of an endocast presents an issue for landmark placement due to the lack of clear surficial unions of tissues or other large features that help define discrete landmarks [17]. We placed evenly spaced 3-D semilandmarks on five sections of the endocast (i.e., left and right cerebrum, left and right optic lobes, and cerebellum). To do this, we first placed discrete Type I or Type II landmarks (sensu [33], also see [34]) along major endocranial features (e.g. the triple point between the optic lobe, cerebrum, and cerebellum). These landmarks were used to define patches to place sliding semilandmarks on each brain section. With this approach, each patch comprises discrete landmarks anchoring the patch boundary, 3-D semilandmarks on lines between discrete landmarks along the surface of the endocast model, and 3-D surface semilandmarks within the patch. Patches of 6 × 6 sliding semilandmarks were used for each half of the cerebrum (Fig. 2, Additional file 1: Tables S3 and S4). 4 × 4 patches were used for each optic lobe. A single patch of 4 × 5 semilandmarks was used for the cerebellum. In places where the edges of neighboring patches overlapped, we manually deleted the duplicated landmarks in R, leaving a total of 109 landmarks. We chose the number of semilandmarks for each patch so as to capture the shape of each lobe without oversampling it, which we confirmed using the function LaSEC in the LaMBDA R package [35]. The raw landmark files are available on Dryad.

The landmark data were imported into R v3.1.1 [36], and aligned using “geomorph” v3.0.3 [37] with generalized Procrustes superimposition and with sliding semilandmarks minimizing total bending energy [15, 16]. The aligned coordinates were imported into MorphoJ v1.06a [38] as two datasets: 1) full sample with non-avian and avian dinosaurs (*N* = 80; ‘Coelurosaur dataset’) and 2) subsampled dataset with the crown-group birds (*N* = 73; ‘Aves dataset’). An analysis using the Coelurosaur dataset indicated that landmark placement on *Alioramus* may have been incorrect, probably due to deformation and lack of easily identifiable landmarks. Therefore, a third dataset was created excluding *Alioramus*, which is discussed below as the ‘Coelurosaur dataset.’ Due to bilateral symmetry, the symmetrical component of the shape data was used for further analyses. Species with multiple specimens were aligned with the pooled data and the mean shape was calculated for each species.

### Analyses

We subjected each shape dataset to a principal components analysis (PCA) in MorphoJ. Plotting the scores associated with the first three PC axes creates a morphospace of the overall shape differences among specimens and identifies the morphological changes occurring along each axis. In addition to endocast images, ‘lollipop’ diagrams were used to visualize major shape changes occurring in morphospace along PC axes, where the vectors indicate the direction and magnitude of change from the mean shape. Using the “geomorph” R package [37], we also created a phylomorphospace based on the first two PC axes. The plot shows the correspondence of phylogenetic relatedness to morphological resemblance and can illustrate morphological innovation through the amount of morphospace explored by taxa [39]. This analysis visualizes unequal magnitude of change per clade or branch and unequal morphological innovation by the geometry and relative length of the branches [39].

A dataset containing the PC axes encompassing 95% of the total shape variation was exported from MorphoJ and imported into R to run a linear discriminant analysis (LDA) using the “MASS” R package v7.3–45 [40]. For the Coelurosaur dataset, we used the first 17 PC axes that encompass 95% of the total shape variation. This analysis looks to maximally separate the a priori locomotor groups (e.g. terrestrial, volant, secondarily flightless). If the LDA is able to adequately separate the shape data into a priori groups, then it suggests that locomotor mode induces certain neuroanatomical shapes across taxonomic groups. If not, then those groups are not supported. Group membership was cross-validated to see if the LD axes are able to correctly predict group assignment. Two locomotor categories (‘protoflying’ and ‘swimming’) had single members and therefore those groups were not included in the dataset for the analysis. The data point for these members were subsequently projected onto the morphospace based on LDA using the ‘predict’ function in R. We also conducted cross-validation analysis to assess the ability to correctly assign locomotory mode based on endocranial shape.

These analyses were run twice. The first analysis was run with crown-group avians (*N* = 73 specimens representing 44 species) and their locomotory mode (e.g. volant or flightless). 'Flightlessness' among extant birds was defined by a complete loss of lift generation, i.e., a total inability to create sufficient lift to raise the body off the ground for any amount of time, such as the ostrich (*Struthio camelus*), dodo (*Raphus cucullatus*), and kakapo (*Strigops habroptilus*) and was labeled ‘flightless’ in these analyses. A penguin, *Eudyptes chrysocome*, is present in the dataset. Penguins use subaqueous flight to propel through the water [41] and therefore it was placed in its own locomotion category (‘swimming’). As it was the only specimen for the ‘swimming’ locomotor category, it was removed from the dataset for LDA. Therefore, the first LDA was performed with two a priori groups: ‘volant’ (*N* = 37 specimens, representing 25 species), and ‘flightless’ (*N* = 35 specimens, representing 18 species), for a total of 72 specimens and 43 species.

The second LDA contained the original set of crown-group birds and six non-avian dinosaurs (three oviraptorosaurs, two troodontids, and *Archaeopteryx*). The non-avian dinosaurs (excluding *Archaeopteryx*) were categorized as ‘terrestrial’ because their primary locomotory mode was not inherited from flying ancestors. The distinction here between ‘secondarily flightless’ and ‘terrestrial’ is important because ‘secondarily flightless’ indicates that the species evolved from flight-capable ancestors, whereas ‘terrestrial’ indicates their ancestors were never capable of volant activity. Although a penguin (*Eudyptes chrysocome*) is present in the dataset, it was again removed for this analysis. Similarly, because of continuing debate concerning the level of flying ability of *Archaeopteryx* [12, 42, 43], it was defined as a ‘proto-flyer’ and was the only member of that locomotor category. As such, *Archaeopteryx* was also removed from the dataset for the LDA. Therefore, this LDA was performed with three a priori groups: ‘terrestrial’ (*N* = 5 specimens, each representing a single species), ‘volant’ (*N* = 37 specimens, representing 25 species), and ‘flightless’ (*N* = 35 specimens, representing 18 species), for a total of 77 specimens and 48 species. Once the analysis was complete, the ‘predict’ function was used to place *Archaeopteryx* and *Eudyptes* into the LD morphospace.

To test explicitly whether locomotory mode and other factors, such as allometry and phylogenetic inertia, drive predictable changes to endocranial shape, we performed several regression analyses. First, regressions of the symmetric component of the shape data against log-transformed centroid size were run for each dataset in MorphoJ using 10,000 replicates to assess the allometric effect. Second, phylogenetically-informed least-squares analyses on locomotory mode and shape data were performed using the ‘procD.pgls’ function in the “geomorph” R package. In addition, we evaluated the effect of phylogenetic signal using the ‘physignal’ function in the “geomorph” R package and allometry based on log-transformed centroid sizes exported from MorphoJ that were used as a proxy for endocranial size [44, 45].

## Results

### Principal components analyses

**Fig. 3:**
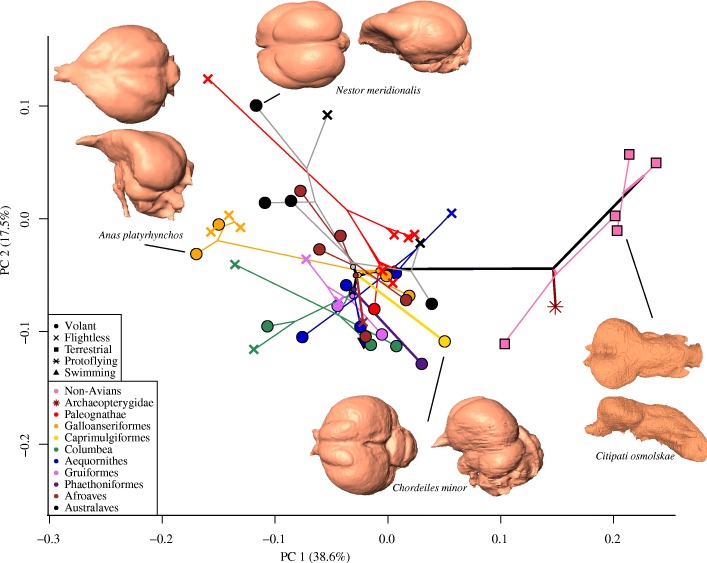
Endocranial shape variation and phylomorphospace for the Coelurosaur dataset. Morphospace constructed from PC1 and PC2 of symmetric component of shape. Symbols by locomotor mode and color-coded taxonomically. Bold, black branches indicate stem Neornithes. Branches leading to clades are color-coded with the same colors, and their hypothetical ancestral shapes are noted with a small circle of the same color

The PCA of the Aves dataset generated 17 PC axes accounting for 95% of the symmetric component of shape variation, with the first three axes associated with 66.6% of the variation (36.4, 16.2, and 13.8%, respectively) (Additional file 1: Figure S1; overall distribution of data points resemble Coelurosaur dataset, Fig. 3). The shape of the posterior aspects of the endocast drive neuroanatomical changes along PC1, where positive scores indicate an anteroventral shift in the posterior margin of the cerebrum, a posteroventral shift in cerebellar location relative to the cerebrum, and a posterior shift and dorsoventral expansion of the optic lobes (Additional file 1: Figure S2B). PC2 correlates with lateral and anterior expansion of the anterior cerebrum, posteroventral reduction of the cerebellum, and a poteromedial shift of the optic lobes (Additional file 1: Figure S2C). Importantly, the plot of PC2 versus PC1 results in no clear visual separation between flying and flightless birds, but rather data points tend to cluster by clade (Fig. 3, Additional file 1: Figure S1). There is variable directionality in shape change within pairs of volant-flightless sister taxa. For example, there is a negative shift along PC1 in Columbiformes (from *Caloenas nicobarica* to *Raphus cucullatus*), but a positive shift along PC1 in Psittaciformes (from *Nestor meridionalis* to *Strigops habroptilus*), and a positive shift in PC2 in Cariamiformes (from *Cariama cristata* to *Pelecyornis australis*) (Additional file 1: Figure S1). In some groups, flightless members radiate in multiple directions away from volant members (e.g. Gruiformes and Anseriformes).

**Fig. 4:**
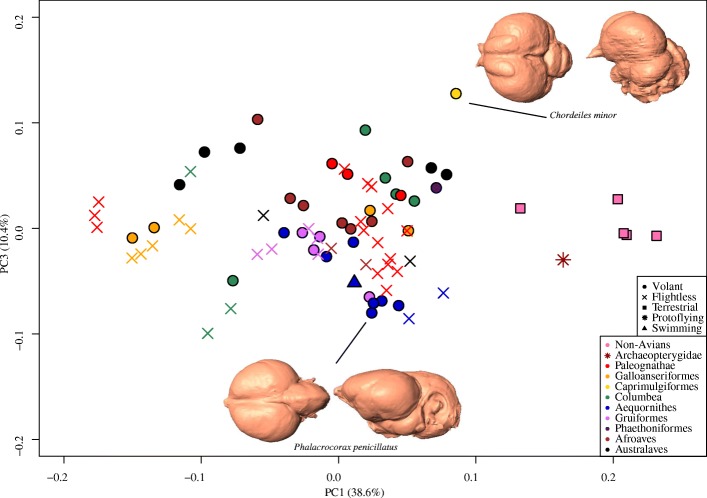
Endocranial shape variation in the Coelurosaur dataset. Morphospace constructed from PC1 and PC3 of symmetric shape component in Coelurosaur dataset, color-coded taxonomically

Within the Coelurosaur dataset the first 17 axes account for 95% of the symmetric component of shape variation, with the first three axes associated with 66.4% of the variation (38.7, 17.5, and 10.4%, respectively) (Fig. 3). The shape changes corresponding to PC1 and PC2 axes are equivalent to those in the Aves dataset. PC1 correlates with a relative anterodorsal shift of the anterior margin of the cerebrum, anteroventral shift of the anterior margin of the cerebellum and relative decrease in dorsoventral height of the cerebrum, and a posteroventral shift of the posterior margin of the cerebellum (Fig. 3 inset endocrania, Additional file 1: Figure S3B). Along PC2, more positive numbers indicate an anteroventral shift in the anterior cerebrum, a lateral reduction of the optic lobes, and a posterior shift and expansion of the cerebellum (Fig. 3 inset endocrania, Additional file 1: Figure S3C). The cumulative effect of these shifts is an anteroposteriorly longer and mediolaterally narrower endocast towards positive PC1 and a shorter, rounder endocast towards negative PC1. The PC morphospace of the Coelurosaur dataset shows no clear visual distinction between modern flying and flightless birds, however non-avian dinosaurs, as well as *Archaeopteryx*, occupy a unique region of morphospace (Fig. 3). Plots of PC3 versus PC1 show some separation between flying and flightless taxa in both the Aves (Additional file 1: Figure S4) and Coelurosaur datasets (Fig. 4), with non-avian dinosaurs again occupying their own morphospace. In both analyses, changes along PC3 are driven primarily by the lateral expansion of the cerebrum, a ventral shift of the anterior margin of the cerebrum, and a dorsolateral shift in the optic lobe (Fig. 4 inset endocrania, Additional file 1: Figure S5).

In phylomorphospace, the early history of the coelurosaurian lineage begins on the positive end of PC1 and shifts negatively (Fig. 3). Along PC1, *Archaeopteryx* occupies a position that lies between non-avian dinosaurs and crown-group birds (Fig. 3). Many internal nodes along the avian stem, as estimated by ancestral state reconstructions, appear in a small section of morphospace, which may be due to poor sampling at the base of Aves. Radiations from this small section of morphospace contain several subclades and their hypothetical ancestors that correspond to the origin of Neoaves. The test for phylogenetic signal indicates that phylogenetic signal is very low, but significant, in both the Aves dataset (Blomberg’s *K* = 0.04; *P* < 0.0001) and the Coelurosaur dataset (*K* = 0.05*; P* < 0.0001).

### Linear discriminant analysis

**Fig. 5:**
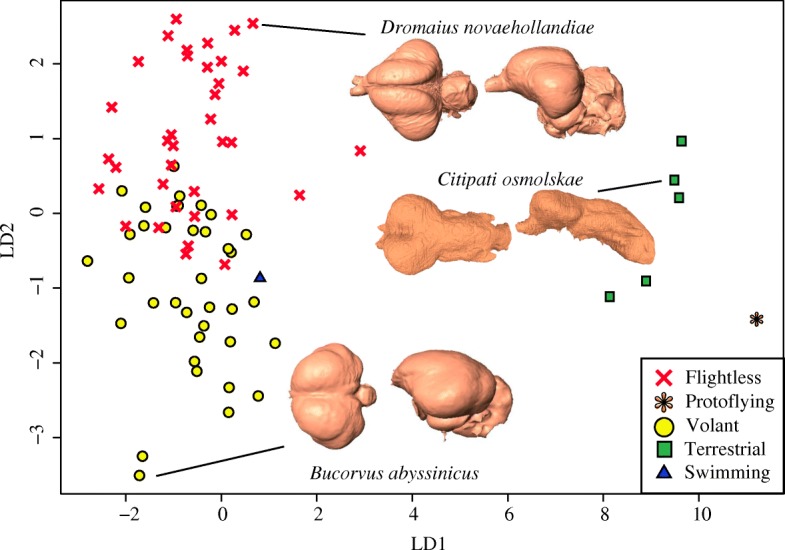
Morphospace constructed from LD1 and LD2 of endocranial shape variation in the Coelurosaur dataset, color-coded by locomotion. Endocasts of representative taxa are shown in dorsal (left) and lateral (right) views

Based on PC axes that account for 95% of symmetric component of shape, the morphospace constructed from LD axes and the Coelurosaur dataset shows crown group birds in a distinct cluster from non-avian dinosaurs and is able to visually distinguish between locomotory modes (Fig. 5). LD1 distinguishes non-avian dinosaurs and crown-group birds. However, volant and flightless crown-group birds are separated along LD2, orthogonal to major neuroanatomical changes along the dinosaur-bird transition. Shape changes towards negative LD2 are anteroposterior contraction of the cerebrum, posterolateral expansion of the cerebrum, narrowing of the cerebellum both in terms of lateral and anteroposterior extent, and an anterior and ventral shift of the optic lobes. Interestingly, the cross-validation technique based on LDA classifies *Archaeopteryx* as terrestrial with a posterior probability of ~1 (neither flying nor secondarily flightless, which had posterior probabilities of 3.89 e^− 30^ and 0.252e^− 29^, respectively). The Rockhopper penguin, *Eudyptes chrysocome*, falls among the volant birds with a posterior probability of 0.853, versus 0.147 for flightless and 2.66e^− 16^ for running. A cross-validation analysis indicated that some taxa were misclassified (Additional file 1: Table S5). *Rallus philippensis* (two of three specimens), *Gallus gallus, Nestor meridionalis, Diomedea immutabilis, Phalacrocorax penicillatus* (all three specimens), *Coragyps atratus*, *Tachyeres patachonicus*, *Anas platyrhynchos*, and *Podilymbus podiceps* are all volant birds that were classified as secondarily flightless. *Phalacrocorax harrisi* (one of two specimens), *Rhea americana* (one of three specimens), *Pelecyornis australis*, *Gallirallus rovianae* (two of three specimens), *Raphus cucullatus*, *Tachyeres pteneres* (one of three specimens), *Strigops habroptilus*, and *Gallirallus australis* (both specimens) are flightless birds that were classified as volant. Lastly, IGM 100/1126, a troodontid, which is terrestrial, was classified as flying. The other 53 specimens were correctly classified by this analysis. The LDA for the Aves dataset (Additional file 1: Figure S6), shows separation along the single LD axis between volant and flightless birds with small overlap in value between the two locomotory modes.

### Covariates

The lack of parallel neuroanatomical changes associated with the dinosaur-bird transition and volant-flightless birds implies that shifts between these locomotory modes incur contrasting changes to brain shape or these shifts have not contributed substantially to neuroanatomical variation. To explicitly assess the impact of locomotory mode on endocranial shape, we tested for the effect of locomotory mode on endocranial shape before and after correcting for phylogenetic structure and allometric signal. The ahistorical analyses indicate that size has a statistically significant effect in both the Aves dataset *(*R^2^ = 0.169; *P* < 0.0001) and the Coelurosaur dataset: (R^2^ = 0.106; *P <* 0.0001). However, when examined in a phylogenetic context, both size and locomotion independently and together failed to reject the null hypothesis of no effect on the shape data for the Aves dataset (*P* > 0.44; Table 1). The Coelurosaur dataset returned significant results for the effect of size (R^2^ = 0.397; *P* = 0.049) and locomotion (R^2^ = 0.007; *P* = 0.004) separately, but not the combined effects of size and locomotion (R^2^ = 0.295; *P* = 0.496; Table 1).

**Table 1:** Results from the phylogenetically informed regression of symmetric component of shape onto locomotory mode and centroid size

Analysis	Variable	Results		
		R^2^	F	P
Aves	Locomotion	0.423	14.69	0.439
	Log Centroid Size	0.497	40.45	0.570
	Locomotion and Log Centroid Size	0.006	2.61	0.678
Coelurosaur	Locomotion	0.007	0.03	<0.001
	Log Centroid Size	0.397	30.90	0.049
	Locomotion and Log Centroid Size	0.295	4.26	0.496

## Discussion

Our study presents several notable results regarding the relationship between flight capacity and brain shape evolution. These results, visually shown by the PC morphospaces, are not congruent with the hypothesis that flightless birds revert to a plesiomorphic brain shape that is similar to their terrestrial, non-avian relatives. In fact, there is comparatively little shape difference between flightless birds and their closest flying relatives (Fig. 3), indicating that flightless birds largely retain brain shapes more similar to their closest living relatives. Moreover, non-volant birds do not collectively approach the endocranial shape of non-avian theropods. Therefore, based on visual inspection of the PC morphospace, the neuroanatomy of flightless birds is clearly not a reverse analogue of flightless non-avian dinosaurs.

The LD morphospace, based on axes that maximize separation between group means, distinguishes between non-avian and avian groups, as expected from PCA. Importantly, it shows an overall separation between volant and flightless birds along LD2 with some overlap (Fig. 5). This shows that some aspects of endocranial shape can distinguish volant and flightless birds. The shape variation associated with LD2 implies that flightless birds, relative to their volant counterparts, generally exhibit anteroposterior expansion of the cerebrum, decreased dorsal convexity of the cerebrum, narrowing of the cerebellum, and inward contraction of the optic lobes.

Meanwhile, cross-validation analysis misclassifies the locomotory mode of approximately 30% of the specimens based on both the Aves and Coelurosaur dataset, indicating that neuroanatomical shape is not as reliable a neuroanatomical correlate for locomotor categories as the LD morphospace would make it seem. Among non-avian dinosaurs, IGM 100/1126 is misclassified as flying (Additional file 1: Table S5). IGM 100/1126 falls closer to the PC morphospace area of crown group birds than the other non-avian dinosaurs and *Archaeopteryx* (Fig. 3), even though it falls within the non-avian morphospace in the LDA (Fig. 5). However, the small number of non-avian coelurosaurs sampled for this study (*N* = 5) may simply have contributed greater degree of uncertainty in estimating locomotory mode. Sampling additional non-avian coelurosaurs through additional discoveries of well-preserved skulls may enhance the capacity of endocranial shape to distinguish between terrestrial and secondarily flightless modes. Importantly, none of the flightless birds were misclassified as terrestrial and based on Aves dataset, the locomotory mode of birds is misclassified for 25 of 72 taxa. This result corroborates the observation from PC morphospace that flightless and volant birds overlap in their neuroanatomical shape, but are distinct from non-avian dinosaurs. Taken together, the results from PC morphospace and LDA imply that although some neuroanatomical differences may be associated with secondary loss of flight, these changes account for very small proportion of shape variation to be a meaningful predictor for locomotory mode.

Furthermore, whereas ahistorical regression analyses on both the Aves and Coelurosaur datasets indicate a significant effect of allometric signal on brain shape, phylogenetically-informed regression analyses indicate that locomotory mode is not a significant predictor of endocranial shape with or excluding allometric signal for the Aves dataset (Table 1). The phylogenetically-informed analysis of variance of the Coelurosaur dataset returned significant results for the independent effect of locomotory mode and size on the shape of the endocast but insignificant results when tested together (Table 1). As such, there is no evidence that locomotory mode is a strong predictor of endocranial shape when corrected for size. Taken together, major shifts in locomotory mode have not driven uniform neuroanatomical changes within crown-group birds. Another possibility explaining the lack of locomotor signal is that the divergence of more recently evolved flying and flightless avian sister taxa occurred on a much shorter time-scale than the dinosaur-bird transition. Consequently, many flightless lineages may not have yet had the time to evolve clear neuroanatomical changes that would be associated with flightlessness. The latter scenario, however, is unlikely for paleognaths, which have been flightless since the early Cenozoic [46].

It is worth noting that the shape changes in this study only reflect surficial differences in neuroanatomy because we employed endocasts. As such, shifts in locomotory mode may have an impact on internal neuroanatomy that cannot be detected through the use of endocasts. Nevertheless, some surficial anatomy may reflect changes occurring internally. Although it cannot be confirmed with the currently available data, the separation of non-avian dinosaurs and crown-group birds in PC morphospaces indicate a clear trend towards relative expansion of the cerebrum along the dinosaur-bird transition (Figs. 3 and 4), which may be related to increased stimulus processing ability [47]. A recent study suggests that the Wulst (a visual and somatosensory integration and processing center) and the entopallium (a visual interpretation area) are important in fast-paced visual processing during flight [5]. Although the landmark scheme used here did not explicitly test for shape differences in the Wulst, the expansion of the posterodorsal region of the cerebrum in avian evolution (Fig. 3, Additional file 1: Figure S2), where these flight-related nuclei lie, may correspond to an increased trend in volant behaviors [5]. Previous studies based on proportional volumes have shown that the cerebral expansion did not appear abruptly at the origin of Avialae, but rather, occurred more gradually throughout coelurosaurian evolutionary history [11, 12]. Inflated, ‘avian-like’ brain volumes first appear at the base of Maniraptora [11] and significant volumetric expansion of the cerebrum does not occur until well within the crown group [12]. However, the shape data here demonstrate that the neuroanatomical shape differences between non-avian dinosaurs and crown-group birds are primarily driven by the cerebrum. Collectively, these results suggest that cerebral shape may have changed in response to the increased use of flight behaviors between non-avian dinosaurs and the modern radiation of birds even as proportional volume remained relatively constant [12]. Intriguingly, LD2, that separates volant and flightless birds, is associated with slight reduction in the convexity of the cerebrum, including the Wulst, suggesting that its relative expansion could reflect flight capacity across coelurosaurs. Such investigation requires explicit characterization of Wulst morphology.

Although the overall predictive power of endocranial shape for locomotory mode is weak, the LDA based on current sampling classifies *Archaeopteryx* as terrestrial, and not volant. This is an intriguing result given the ongoing debate about its flight capabilities [42, 43]. The endocast of *Archaeopteryx* seems to more closely resemble its non-avian relatives rather than crown group birds, meaning it may not have been capable of extensive volant behaviors. Increased taxonomic sampling that fills in the gap between *Archaeopteryx* and the modern radiation of birds may strengthen our ability to classify the locomotory mode of the earliest members of Aves. The LDA classifies *Eudyptes* as a volant bird. Early studies note that the penguin uses subaqueous flight for locomoting underwater [41], and our study shows that its neuroanatomical shape corroborates the notion that subaqueous flight is equivalent to aerial flight. This outcome may be due to the fact that the evolution of wing-propelled diving (i.e. the acquisition of a new behavior) may have played a larger role in shaping the brain than the initial loss of aerial flight (i.e. the loss of an established behavior) [48].

If not locomotory mode, then what are the major drivers of brain shape evolution in birds? Previous GM studies on avian endocasts have shown that phylogenetic history, allometry, and orbit shape account for statistically significant (*P* < 0.001), but small proportions of endocranial shape variation [17, 49]. With different taxonomic sampling and use of high-dimensional shape characterization, our results corroborate these findings, indicating that there is allometric signal and a very weak, albeit statistically significant, phylogenetic signal present in the endocranial shape datasets. While these factors have variably contributed to evolutionary changes in bird brains, they collectively account for a small proportion (~ 10%) of the total variation [e.g., 49], suggesting that additional drivers are yet to be identified [49].

In addition, increased taxonomic sampling is still needed between the base of Avialae and the origin of living birds. Unfortunately, most of the fossils from this interval are fragmentary [50] or crushed [51] making endocranial construction extremely difficult. This paucity of specimens contributes to the apparent gap between *Archaeopteryx* and modern birds in the phylomorphospace, preventing a more reliable model of neuroanatomical evolution along this lineage. Early avialans undertook a variety of locomotor strategies, from the fully volant Enantiornithines [52] to the swimming Hesperornithiformes [53]. Sampling this region of the tree, perhaps from recently discovered specimens [54], might bridge the gap from the non-volant theropods and at least partially volant *Archaeopteryx* to the fully volant crown group and provide examples of transitional brain morphologies not represented by living or extinct theropods.

## Conclusions

The osteological changes associated with the evolution of flight in theropod dinosaurs is well documented in the fossil record, but the accompanying neuroanatomical changes are less known. Here, we used 3-D GM techniques on endocasts of extinct and modern dinosaurs (birds) to quantify and evaluate neuroanatomical changes related to this major locomotory change. In particular, we analyzed closely-related sets of volant and flightless birds to use as a potential reverse analogue to the initial acquisition of flight. The results demonstrate that loss of flight capacity does not incur predictive changes to endocranial shape in extant birds. In addition, the brain morphology of flightless birds do not converge to that of non-avian dinosaurs. While additional sampling may close the gap between non-avian dinosaurs and Neornithes, brain evolution from non-avian dinosaurs to birds appears to have been a unidirectional phenomenon or “point of no return.” Among crown-group birds, returning to an ancestral locomotory mode (i.e., flightlessness) does not correlate with a reversion to the more pleisiomorphic brain shape found in terrestrial non-avian dinosaurs. Modern flightless birds, therefore, are not reverse neuroanatomical analogues of non-avian dinosaurs.

## Additional file

Additional file 1: **Table S1.** CT scanning parameters used for each specimen scanned for this project. **Table S2.** CT scanning parameters used for additional specimens used for this project from Balanoff et al. [11]. **Table S3.** Discrete landmarks and anatomical descriptions. **Table S4.** Sliding semilandmarks used and anatomical descriptions. **Table S5.** Posterior probabilities from the cross-validation analysis of the LDA of the Coelurosaur dataset. **Figure S1.** Endocranial shape variation in the Aves dataset, illustrated in a morphospace constructed from PC1 and PC2. **Figure S2.** Shape changes along positive PC1 and PC2 for the Aves dataset shown as lollipop diagrams in dorsal and right lateral views. **Figure S3.** Shape changes along positive PC1 and PC2 for the Coelurosaur dataset shown as lollipop diagrams in dorsal and right lateral views. **Figure S4.** Endocranial shape variation in the Aves dataset, illustrated in a morphospace constructed from PC1 and PC3. **Figure S5.** Shape changes along positive PC3 for the Aves and Coelurosaur datasets shown as lollipop diagrams in dorsal and right lateral views. **Figure S6.** Bar plot of the single linear discriminant axis constructed from the LDA of endocranial shape variation in the Aves dataset. (DOCX 1520 kb)

## Abbreviations

AMNH (FARB): American Museum of Natural History, New York, NY, USA (Fossil Amphibian, Reptile, and Bird Collection)
FMNH: Field Museum of Natural History, Chicago, IL, USA
IGM: Mongolian Institute of Geology, Ulaanbaatar, Mongolia
IVPP: Institute of Vertebrate Paleontology and Paleoanthropology Beijing, People’s Republic of China
KU: University of Kansas Natural History Museum, Lawrence, KS, USA
NHMUK (PV OR, A): Natural History Museum, London, United Kingdom (Paleontology Vertebrates and Old Register, Aves)
NMNH: Smithsonian Institution National Museum of Natural History
TCWC: Texas Cooperative Wildlife Collection, College Station, Texas, USA
TMM: University of Texas Memorial Museum, Austin, Texas, USA
