# Supplementary material for: Flightless birds are not neuroanatomical analogs of non-avian dinosaurs
Source: BMC Evol Biol. 2018 Dec 13;18:190. doi: 10.1186/s12862-018-1312-0 (PMC6293530; doi:10.1186/s12862-018-1312-0)
Supplement: Supplementary file 1 — Table S1. CT scanning parameters used for each specimen scanned for this project. Table S2. CT scanning parameters used for additional specimens used for this project from Balanoff et al. [11]. Table S3. Discrete landmarks and anatomical descriptions. Table S4. Sliding semilandmarks used and anatomical descriptions. Table S5. Posterior probabilities from the cross-validation analysis of the LDA of the Coelurosaur dataset. Figure S1. Endocranial shape variation in the Aves dataset, illustrated in a morphospace constructed from PC1 and PC2. Figure S2. Shape changes along positive PC1 and PC2 for the Aves dataset shown as lollipop diagrams in dorsal and right lateral views. Figure S3. Shape changes along positive PC1 and PC2 for the Coelurosaur dataset shown as lollipop diagrams in dorsal and right lateral views. Figure S4. Endocranial shape variation in the Aves dataset, illustrated in a morphospace constructed from PC1 and PC3. Figure S5. Shape changes along positive PC3 for the Aves and Coelurosaur datasets shown as lollipop diagrams in dorsal and right lateral views. Figure S6. Bar plot of the single linear discriminant axis constructed from the LDA of endocranial shape variation in the Aves dataset. (DOCX 1520 kb) [file 12862_2018_1312_MOESM1_ESM.docx]

Supplementary Information

Table S1: CT scanning parameters used for each specimen scanned for this project.

| Species | Specimen | Voltage (kV) | Current (μA) | Resolution (μm^3^) |
| --- | --- | --- | --- | --- |
| *Alca torda* | AMNH 17532 | 100 | 160 | 100.935 |
|  | AMNH 27816 | 100 | 160 | 100.935 |
|  | AMNH 5977 | 100 | 160 | 94.484 |
| *Apteryx australis* | AMNH 4437 | 160 | 110 | 56.231 |
|  | AMNH 5372 | 160 | 110 | 48.0 |
| *Caloenas nicobarica* | NMNH 19709 | 120 | 170 | 38 |
|  | NMNH 19715 | 120 | 170 | 38 |
|  | NMNH 318506 | 130 | 160 | 41.16 |
| *Cariama cristata* | AMNH 8604 | 180 | 250 | 70.195 |
|  | AMNH 8667 | 180 | 250 | 70.195 |
| *Casuaraius casuarius becarii* | AMNH 3870 | 150 | 130 | 134.28 |
| *Casuarius casuarius* | AMNH 3200 | 150 | 130 | 117.28 |
| *Casuraius unappendiculatus* | AMNH 1553 | 150 | 130 | 108.66 |
|  | AMNH 2729 | 150 | 130 | 83.62 |
| *Crypturellus tataupa* | AMNH 604 | 150 | 130 | 58.56 |
|  | AMNH 605 | 150 | 130 | 58.56 |
|  | AMNH 8560 | 150 | 130 | 50.25 |
| *Dromaius novaehollandiae* | AMNH 11709 | 160 | 110 | 79.6 |
|  | AMNH 18458 | 160 | 110 | 90.9 |
|  | AMNH 3742 | 160 | 110 | 90.9 |
| *Gallirallus australis* | AMNH 19022 | 120 | 150 | 38.55 |
|  | NMNH 19021 | 120 | 170 | 38 |
| *Gallirallus rovianae* | AMNH 28014 | 100 | 180 | 89.08 |
|  | AMNH 30316 | 100 | 170 | 91.17 |
|  | AMNH 30329 | 100 | 180 | 84.52 |
| *Gallus gallus* | AMNH 18555 | 130 | 110 | 79.44 |
| *Nestor meridionalis* | AMNH 27323 | 100 | 160 | 85.60 |
| *Pelecyornis australis* | AMNH 9275 | 220 | 250 | 71.56 |
| *Phalacrocorax harrisi* | AMNH 2312120 | 120 | 160 | 76.41 |
|  | AMNH 4053 | 120 | 160 | 76.41 |
| *Phalacrocorax penicillatus* | AMNH 26482 | 120 | 160 | 73.19 |
|  | AMNH 27350 | 120 | 160 | 76.41 |
|  | AMNH 4876 | 120 | 160 | 77.76 |
| *Pinguinus impennis** | AMNH 260 | 100 | 170 | 85.99 |
|  | AMNH 261 | 100 | 170 | 76.37 |
| *Podylimbus gigas* | NMNH 343470 | 120 | 170 | 38 |
|  | NMNH 343471 | 120 | 150 | 41.16 |
| *Rallus philippensis* | AMNH 2682 | 100 | 160 | 71.84 |
|  | AMNH 3442 | 100 | 170 | 51.38 |
|  | AMNH 3545 | 100 | 160 | 71.84 |
| *Raphus cucullatus* | NHMUK PV A9040 | 210 | 150 | 114.0 |
| *Rhea americana* | AMNH 3783 | 150 | 130 | 82.04 |
|  | AMNH 4090 | 150 | 130 | 80.90 |
|  | AMNH 6470 | 150 | 130 | 90.6 |
| *Strigops habroptilus* | AMNH 2079 | 120 | 160 | 85.95 |
| *Struthio camelus* | AMNH 2775 | 160 | 90 | 80.53 |
| *Struthio molybdophanes* | AMNH 1504 | 160 | 90 | 72.54 |
| *Sturnus vulgaris* | AMNH 21224 | 160 | 90 | 54.47 |
| *Tachyeres brachypterus* | NMNH 555469 | 200 | 240 | 74.88 |
| *Tachyeres leucocephalus* | AMNH 8513 | 150 | 130 | 83.62 |
| *Tachyeres patachonicus* | NMNH 49014 | 200 | 240 | 78.38 |
| *Tachyeres pteneres* | AMNH 1222 | 150 | 130 | 83.62 |
|  | NMNH 490937 | 120 | 170 | 64.8 |
|  | NMNH 490939 | 200 | 240 | 78.38 |

*These specimens are composites and the specimen number refers to the number on their beak.

Table S2: CT scanning parameters used for additional specimens used for this project from Balanoff et al. [11].

| Taxon | Specimen  # | Scanning  Facility | z-spacing (mm) | x-, y-spacing (mm) | Number of slices through skull |
| --- | --- | --- | --- | --- | --- |
| *Anas platyrhynchos* | TMM M-uncat. | UTCT | 0.400 | 0.122 | 123 |
| *Apteryx australis* | AMNH 18456 | UTCT | 0.16 | 0.16 | 983 |
| *Brotogeris chrysopteris* | FMNH 330249 | UTCT | 0.163 | 0.066 | 123 |
| *Bucorvus abyssinicus* | TMM M-5946 | UTCT | 0.263 | 0.117 | 143 |
| *Chauna chavaria* | KU 81969 | UTCT | 0.295 | 0.102 | 115 |
| *Chordeiles minor*  | TMM M-uncat. | UTCT | 0.059 | 0.054 | 381 |
| *Coragyps atratus* | TMM M-uncat. | UTCT | 0.121 | 0.055 | 300 |
| *Eudyptes chrysocome* | TMM M-uncat. | AMNH | 0.104 | 0.104 | 1100 |
| *Fregata magnificens* | FMNH 37858 | UTCT | 0.128 | 0.059 | 269 |
| *Gavia immer* | TCWC 13.300 | UTCT | 0.109 | 0.051 | 408 |
| *Grus canadensis* | TMM M-uncat. | UTCT | 0.166 | 0.076 | 246 |
| *Haliaeetus leucocephalus* | TMM M-7260 | UTCT | 0.169 | 0.077 | 245 |
| *Melanerpes aurifron* | FMNH 108742 | UTCT | 0.060 | 0.052 | 311 |
| *Phaethon rubricada* | FMNH 346039 | UTCT | 0.174 | 0.051 | 171 |
| *Phalacrocorax penicillatus* | TMM M-1180 | UTCT | 0.115 | 0.049 | 399 |
| *Phoebastria immutabilis* | FMNH 313780 | UTCT | 0.138 | 0.063 | 313 |
| *Podilymbus podiceps* | TMM M-7139 | UTCT | 0.056 | 0.026 | 379 |
| *Ptilinopus melanospilus* | TMM M-uncat. | UTCT | 0.121 | 0.056 | 290 |
| *Struthio camelus* | AMNH 3199 | SUNY | 0.310 | 0.250 | 209 |
| *Incisivosaurus gauthieri* | IVPP V 13326 | SUNY | 0.310 | 0.187 | 147 |
| *Citipati osmolskae* | IGM 100/978 | UTCT | 0.250 | 0.196 | 360 |
| *Khaan mckennai* | IGM 100/973 | UTCT | 0.164 | 0.076 | 328 |
| *Zanabazar junior* | IGM 100/1 | UTCT | 0.450 | 0.230 | 195 |
| Unnamed troodontid | IGM 100/1126 | UTCT | 0.047 | 0.043 | 1710 |
| *Archaeopteryx lithographica* | NHMUK PV OR 37001 | UTCT | 0.023 | 0.020 | 996 |
| *Alioramus altai* | IGM 100/1844 | UTCT | 0.250 | 0.294 | 490 |

Table S3: Discrete landmarks and anatomical descriptions (refer to Fig. 2). Landmark numbers in parentheses indicate a symmetrical pair.

| Landmark Number | Anatomical Description |
| --- | --- |
| 6 | Intersection of the cerebrum and cerebellum along the midline |
| 36 | Intersection of the nasal lobes and cerebrum along the midline |
| (31, 74) | Intersection of the anterodorsal point of the optic lobes and the cerebrum |
| (46, 88) | Intersection of the ventral optic lobes and brainstem |
| (1, 49) | Intersection of the optic lobes, cerebrum, and cerebellum |
| (94, 109) | Lateral extent of the anterior cerebellum |
| (91, 106) | Lateral midpoints around the foramen magnum |
| 99 | Dorsal midpoint of the foramen magnum |

Table S4: Sliding semilandmarks used and anatomical descriptions (refer to Fig. 2). Landmarks may be listed more than once to account for being in between multiple endocranial regions. Landmark numbers in parentheses indicate a symmetrical pair.

| Landmark Number | Anatomical Description |
| --- | --- |
| 12, 18, 24, 30 | Midline landmarks in between cerebral lobes |
| (8, 55), (9, 56), (10, 57), (11, 58), (14, 60), (15, 61), (16, 62), (17, 63), (20, 65), (21, 66), (22, 67), (23, 68), (26, 70), (27, 71), (28, 72), (29,73), (32, 75), (33, 76), (34, 77), (35, 78) | Cerebral hemisphere landmarks |
| (2, 50), (3, 51), (4, 52), (5, 53) | Border between cerebellum and cerebrum |
| (92, 107), (93, 108), (95, 102), (96, 103), (97, 104), (98, 105), 100, 101 | Landmarks on the cerebellum |
| (37, 79), (38, 80), (39, 81), (40, 82), (41, 83), (42, 84), (43, 85), (44, 86), (45, 87), (47, 89), (48, 90), | Landmarks on the optic lobe |
| (7, 54), (13, 59), (19, 64), (25, 69) | Border between optic lobe and cerebrum |

Table S5: Posterior probabilities from the cross-validation analysis of the LDA. Bold values indicate their locomotor classification. Bold and italicized numbers indicate an incorrect classification. Asterisks by specimen names refer to specimens whose posteriors probabilities were predicted by the analysis.

| Specimen | Flightless | Flying | Running |
| --- | --- | --- | --- |
| *Alca torda* (AMNH 17532) | 3.19E-02 | **9.68E-01** | 1.31E-21 |
| *Alca torda* (AMNH 27816) | 9.19E-02 | **9.08E-01** | 7.32E-24 |
| *Alca torda* (AMNH 5977) | 7.91E-02 | **9.21E-01** | 3.43E-26 |
| *Anas platyrhynchos* | **8.69E-01** | 1.31E-01 | 1.68E-19 |
| *Apteryx australis* | **9.96E-01** | 3.64E-03 | 5.04E-25 |
| *Apteryx australis* (AMNH 4437) | **9.93E-01** | 7.43E-03 | 8.69E-26 |
| *Apteryx australis* (AMNH 5372) | **9.92E-01** | 8.19E-03 | 7.82E-21 |
| *Archaeopteryx lithographica** | 3.89E-30 | 2.52E-29 | **1.00E+00** |
| *Brotogeris chrysopteris* | 4.98E-02 | **9.50E-01** | 6.03E-21 |
| *Bucorvus abyssinicus* | 1.13E-05 | **1.00E+00** | 8.45E-34 |
| *Caloenas nicobarica* (NMNH 19709) | 7.16E-03 | **9.93E-01** | 3.93E-19 |
| *Caloenas nicobarica* (NMNH 19715) | 3.10E-02 | **9.69E-01** | 1.61E-18 |
| *Caloenas nicobarica* (NMNH 318506) | 2.81E-03 | **9.97E-01** | 6.17E-19 |
| *Cariama cristata* (AMNH 8604) | 7.60E-02 | **9.24E-01** | 1.38E-18 |
| *Cariama cristata* (AMNH 8667) | 3.35E-02 | **9.67E-01** | 1.96E-14 |
| *Casuarius casuarius becarii* (AMNH 3870) | **9.12E-01** | 8.83E-02 | 1.16E-31 |
| *Casuarius casuarius* (AMNH 3200) | **9.95E-01** | 4.98E-03 | 3.34E-18 |
| *Casuarius unappendiculatus* (AMNH 1553) | **7.78E-01** | 2.22E-01 | 7.06E-25 |
| *Casuarius unappendiculatus* (AMNH 2729) | **9.64E-01** | 3.64E-02 | 2.00E-19 |
| *Chauna chavaria* | 3.97E-01 | **6.03E-01** | 2.16E-18 |
| *Chordeiles minor* | 3.75E-03 | **9.96E-01** | 1.44E-13 |
| *Citipati osmolskae* | 2.31E-16 | 1.31E-17 | **1.00E+00** |
| *Coragyps atratus* | **7.24E-01** | 2.76E-01 | 4.73E-21 |
| *Crypturellus tataupa* (AMNH 604) | 2.29E-01 | **7.71E-01** | 7.30E-21 |
| *Crypturellus tataupa* (AMNH 605) | 3.69E-01 | **6.31E-01** | 3.76E-18 |
| *Crypturellus tataupa* (AMNH 8560) | 8.37E-02 | **9.16E-01** | 1.21E-22 |
| *Diomedea immutabilis* | **6.67E-01** | 3.33E-01 | 2.78E-20 |
| *Dromaius novaehollandiae* (AMNH 11709) | **9.80E-01** | 2.01E-02 | 4.16E-21 |
| *Dromaius novaehollandiae* (AMNH 18458) | **9.96E-01** | 3.56E-03 | 3.16E-16 |
| *Dromaius novaehollandiae* (AMNH 3742) | **9.80E-01** | 1.99E-02 | 9.92E-29 |
| *Eudyptes chrysocome** | 1.47E-01 | **8.53E-01** | 2.66E-16 |
| *Fregata magnificens* | 2.85E-01 | **7.15E-01** | 2.84E-35 |
| *Gallirallus australis* (NMNH 19021) | 2.64E-01 | **7.36E-01** | 2.10E-25 |
| *Gallirallus australis* (NMNH 19022) | **6.25E-01** | 3.75E-01 | 1.55E-30 |
| *Gallirallus rovianae* (AMNH 28014) | 2.73E-01 | **7.27E-01** | 8.86E-22 |
| *Gallirallus rovianae* (AMNH 30316) | 1.39E-01 | **8.61E-01** | 1.66E-22 |
| *Gallirallus rovianae* (AMNH 30329) | **6.69E-01** | 3.31E-01 | 4.04E-24 |
| *Gallus gallus* (AMNH 18555) | **8.54E-01** | 1.46E-01 | 4.09E-28 |
| *Gavia immer* | 1.96E-01 | **8.04E-01** | 1.36E-29 |
| *Grus canadensis* | 1.51E-01 | **8.49E-01** | 4.87E-15 |
| *Haliaeetus leucocephalus* | 4.47E-02 | **9.55E-01** | 9.87E-31 |
| IGM100 1126 | 6.99E-03 | **9.93E-01** | 2.79E-08 |
| *Incisivosaurus gauthieri* | 6.17E-10 | 1.28E-08 | **1.00E+00** |
| *Khaan mckennai* | 1.73E-17 | 2.48E-18 | **1.00E+00** |
| *Melanerpes aurifrons* | 1.49E-02 | **9.85E-01** | 6.48E-22 |
| *Nestor meridionalis* (AMNH 27323) | **7.09E-01** | 2.91E-01 | 4.50E-28 |
| *Pelecyornis australis* (AMNH 9275) | 4.81E-01 | **5.19E-01** | 8.30E-22 |
| *Phaethon rubricauda* | 2.36E-04 | **1.00E+00** | 5.52E-30 |
| *Phalacrocorax harrisi* (AMNH 2312) | 3.64E-01 | **6.36E-01** | 2.06E-11 |
| *Phalacrocorax harrisi* (AMNH 4053) | **7.17E-01** | 2.83E-01 | 1.32E-05 |
| *Phalacrocorax penicillatus* | 4.80E-01 | **5.20E-01** | 5.06E-17 |
| *Phalacrocorax penicillatus* (AMNH 26482) | **7.42E-01** | 2.58E-01 | 2.73E-23 |
| *Phalacrocorax penicillatus* (AMNH 27350) | **5.32E-01** | 4.68E-01 | 9.17E-25 |
| *Phalacrocorax penicillatus* (AMNH 4876) | **6.61E-01** | 3.39E-01 | 2.15E-23 |
| *Pinguinus impennis* (AMNH 260) | 3.35E-02 | **9.67E-01** | 1.09E-18 |
| *Pinguinus impennis* (AMNH 261) | 9.79E-02 | **9.02E-01** | 7.86E-23 |
| *Podilymbus gigas* (NMNH 343470) | **7.64E-01** | 2.36E-01 | 1.21E-24 |
| *Podilymbus gigas* (NMNH 343471) | **5.10E-01** | 4.90E-01 | 2.59E-33 |
| *Podilymbus podiceps* | **7.42E-01** | 2.58E-01 | 9.16E-30 |
| *Ptilinopus melanospilus* | 1.08E-02 | **9.89E-01** | 5.49E-22 |
| *Rallus philippensis* (AMNH 2682) | **6.79E-01** | 3.21E-01 | 1.51E-23 |
| *Rallus philippensis* (AMNH 3442) | 4.25E-01 | **5.75E-01** | 1.01E-28 |
| *Rallus philippensis* (AMNH 3545) | **5.69E-01** | 4.31E-01 | 8.89E-22 |
| *Raphus cucullatus* (NHMUKPVA9040) | 8.94E-02 | **9.11E-01** | 9.27E-31 |
| *Rhea americana* (AMNH 3783) | 4.74E-01 | **5.26E-01** | 3.36E-25 |
| *Rhea americana* (AMNH 4090) | **7.96E-01** | 2.04E-01 | 6.19E-19 |
| *Rhea americana* (AMNH 6470) | **7.91E-01** | 2.09E-01 | 4.06E-24 |
| *Strigops habroptilus* (AMNH 2079) | 1.29E-01 | **8.71E-01** | 2.27E-17 |
| *Struthio camelus* | **9.84E-01** | 1.64E-02 | 4.74E-19 |
| *Struthio camelus* (AMNH 2775) | **9.85E-01** | 1.45E-02 | 3.47E-23 |
| *Struthio molybdophanes* (AMNH 1504) | **9.88E-01** | 1.21E-02 | 2.56E-23 |
| *Sturnus vulgaris* (AMNH 21224) | 1.32E-01 | **8.68E-01** | 1.40E-19 |
| *Tachyeres brachypterus* (NMNH 555468) | **9.11E-01** | 8.93E-02 | 1.43E-20 |
| *Tachyeres leucocephalus* (AMNH 8513) | **7.83E-01** | 2.17E-01 | 6.04E-18 |
| *Tachyeres patachonicus* (NMNH 49014) | **9.06E-01** | 9.36E-02 | 3.85E-24 |
| *Tachyeres pteneres* (AMNH 1222) | 1.04E-01 | **8.96E-01** | 2.22E-37 |
| *Tachyeres pteneres* (NMNH 490937) | **9.53E-01** | 4.71E-02 | 4.01E-20 |
| *Tachyeres pteneres* (NMNH 490939) | **9.79E-01** | 2.11E-02 | 1.95E-17 |
| *Zanabazar junior* | 1.56E-15 | 4.74E-18 | **1.00E+00** |


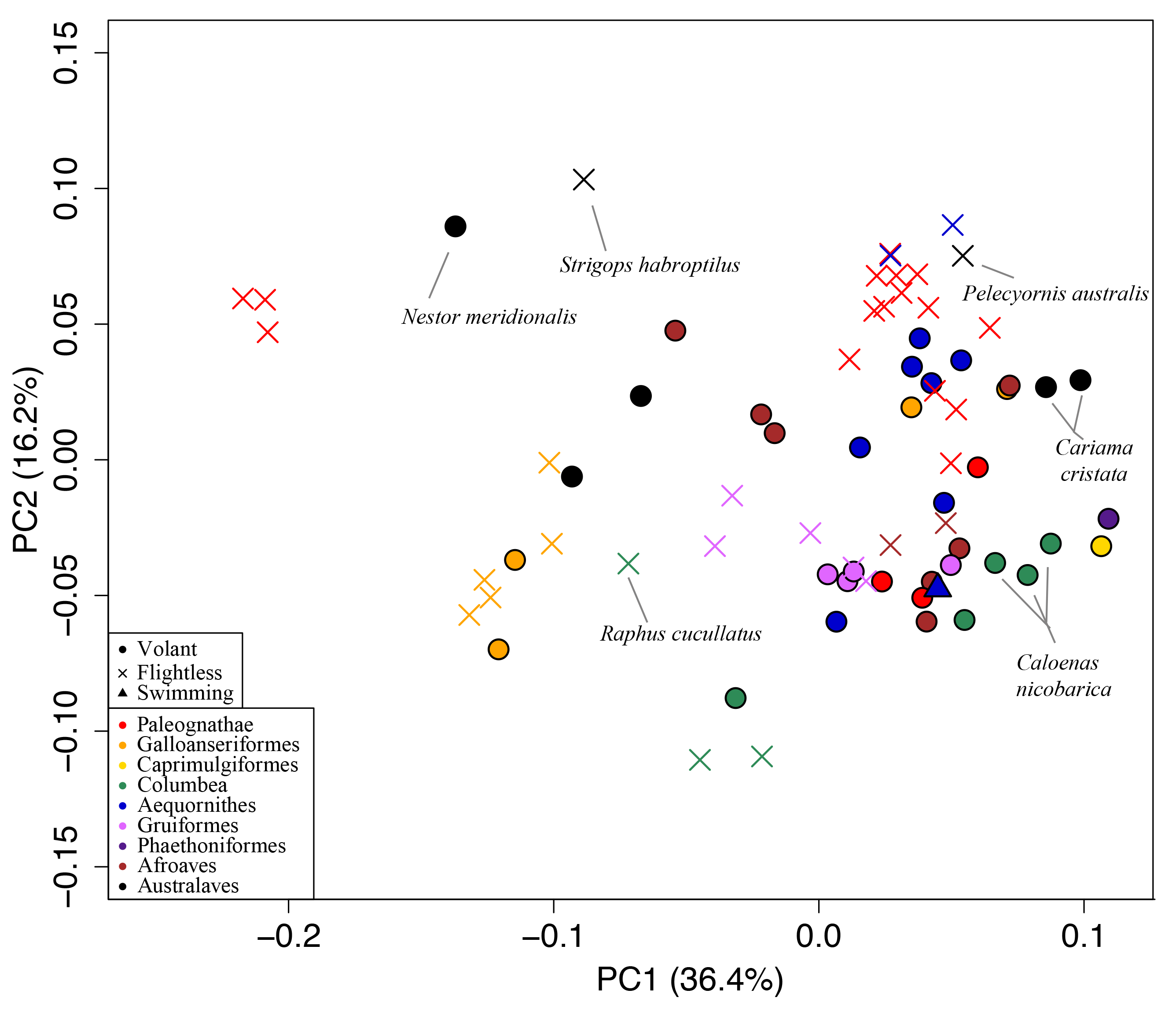


Figure S1: Endocranial shape variation in the Aves dataset. Morphospace constructed from PC1 and PC2 of symmetric component of shape. Symbols by locomotor mode and color-coded taxonomically. Labeled species show variable directionality in shape change within pairs of volant-flightless sister taxa.


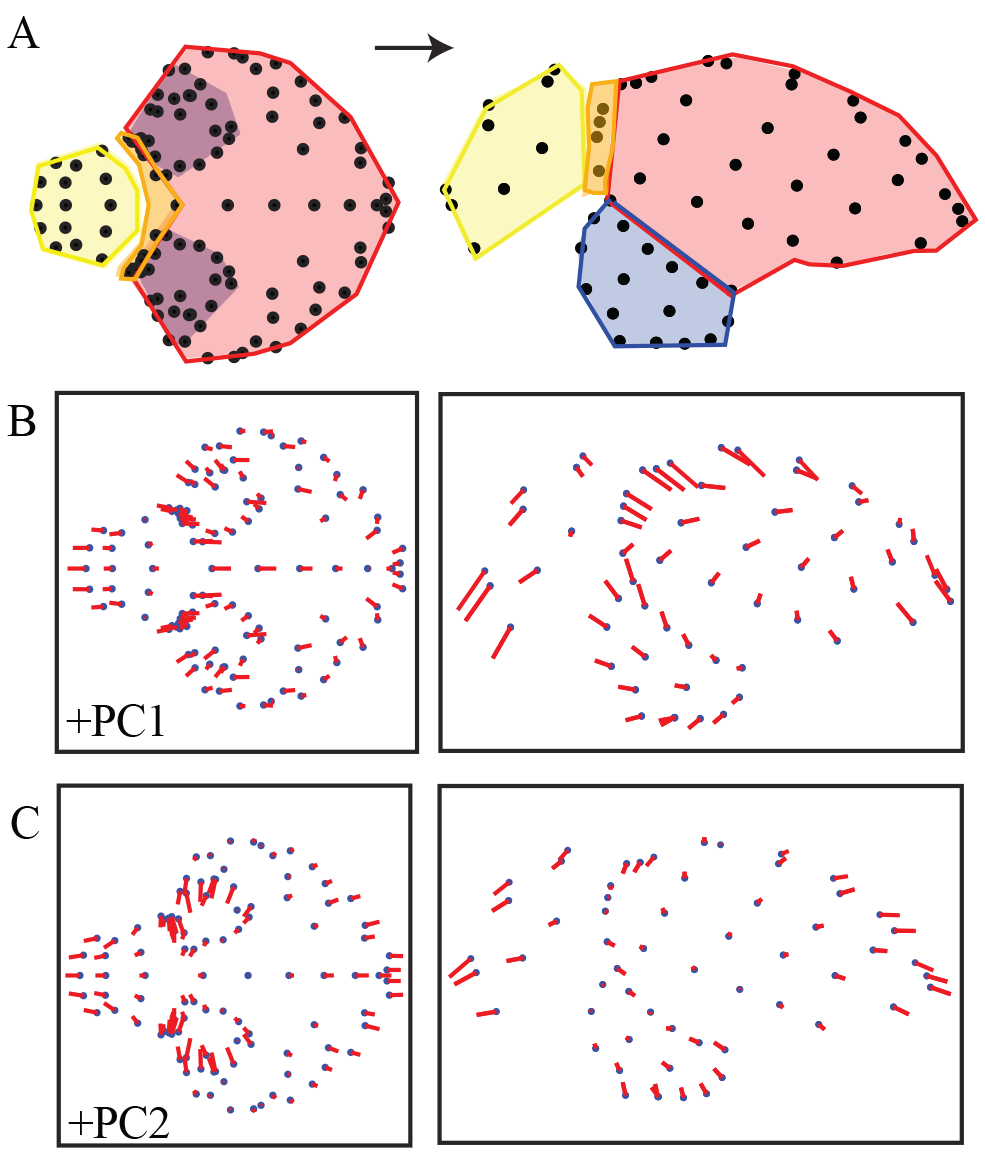


Figure S2: Shape changes along B) positive PC1 and C) positive PC2 for the Aves dataset in dorsal (left) and right lateral (right) views. A) Color-coded key of endocasts regions: Red - cerebrum, orange – intersection of cerebrum and cerebellum, yellow – cerebellum, blue – optic lobe. Dots represent mean landmark positions, and red lines in B and C represent the direction and magnitude of change at the maximum positive range of the specimens on PC1 and PC2. Arrow indicates anterior.


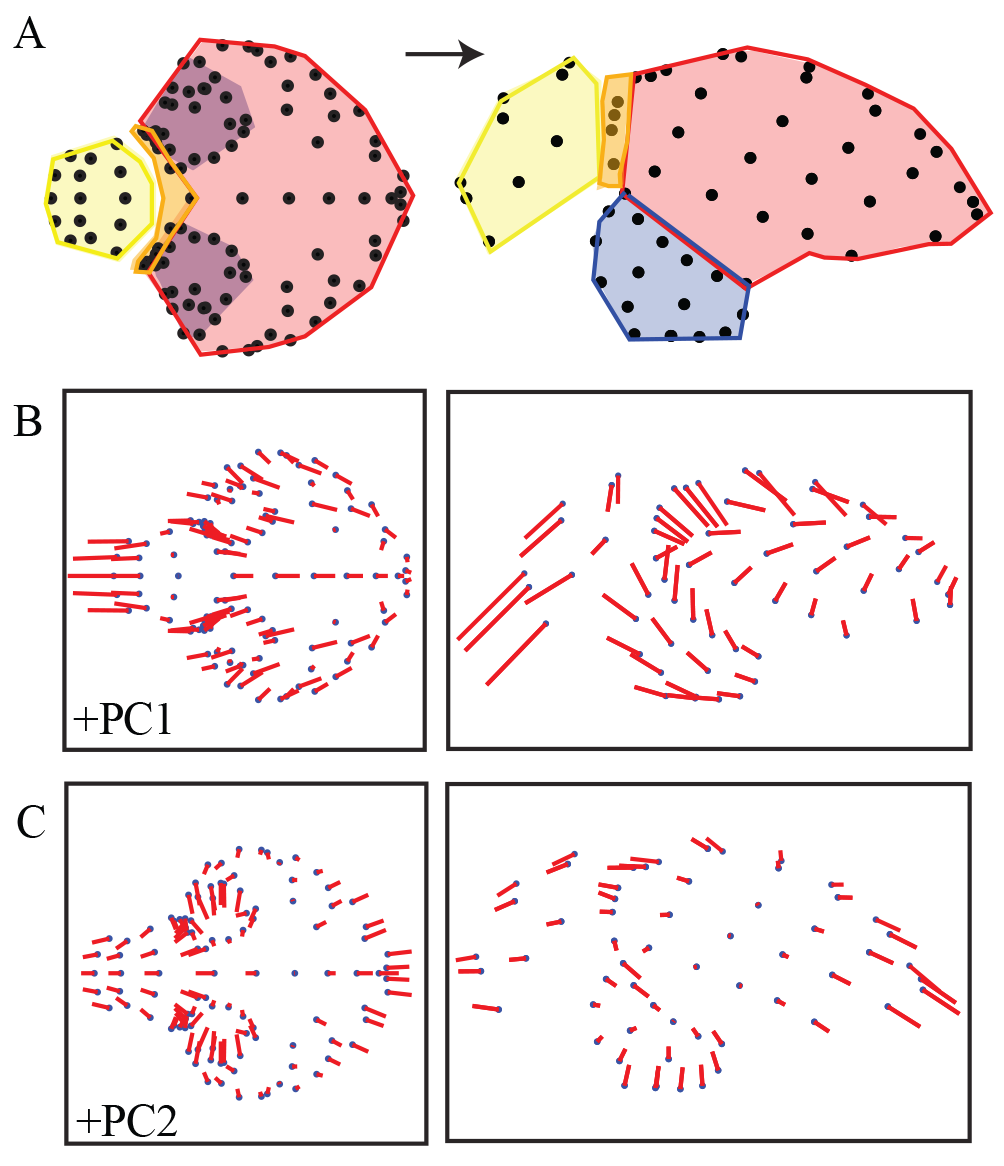


Figure S3: Shape changes along B) positive PC1and C) positive PC2 for the Coelurosaur dataset in dorsal (left) and right lateral (right) views. A) Color-coded key of endocasts regions: Red - cerebrum, orange – intersection of cerebrum and cerebellum, yellow – cerebellum, blue – optic lobe. Dots represent mean landmark positions, and red lines in B and C represent the direction and at the maximum positive range of the specimens on PC1 and PC2. Arrow indicates anterior. Note equivalent shape changes associated with PC1 and PC2 in the Coelurosaur dataset with the opposing sign of the axes (i.e., positive PC1 direction here is negative PC1in Coelurosaur dataset).


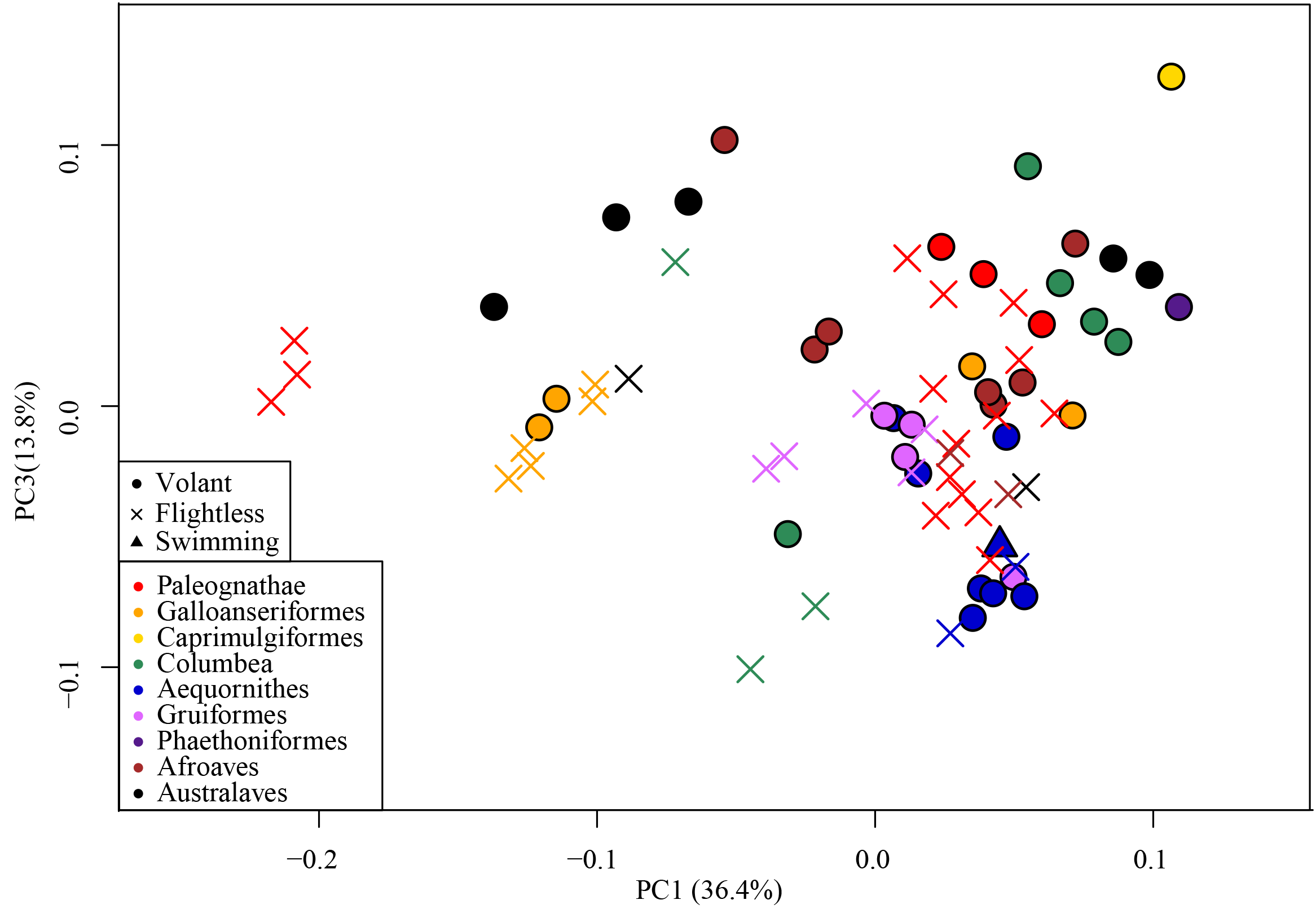


Figure S4: Endocranial shape variation in the Aves dataset. Morphospace constructed from PC1 and PC3 of symmetric shape component in Aves dataset, color-coded taxonomically.


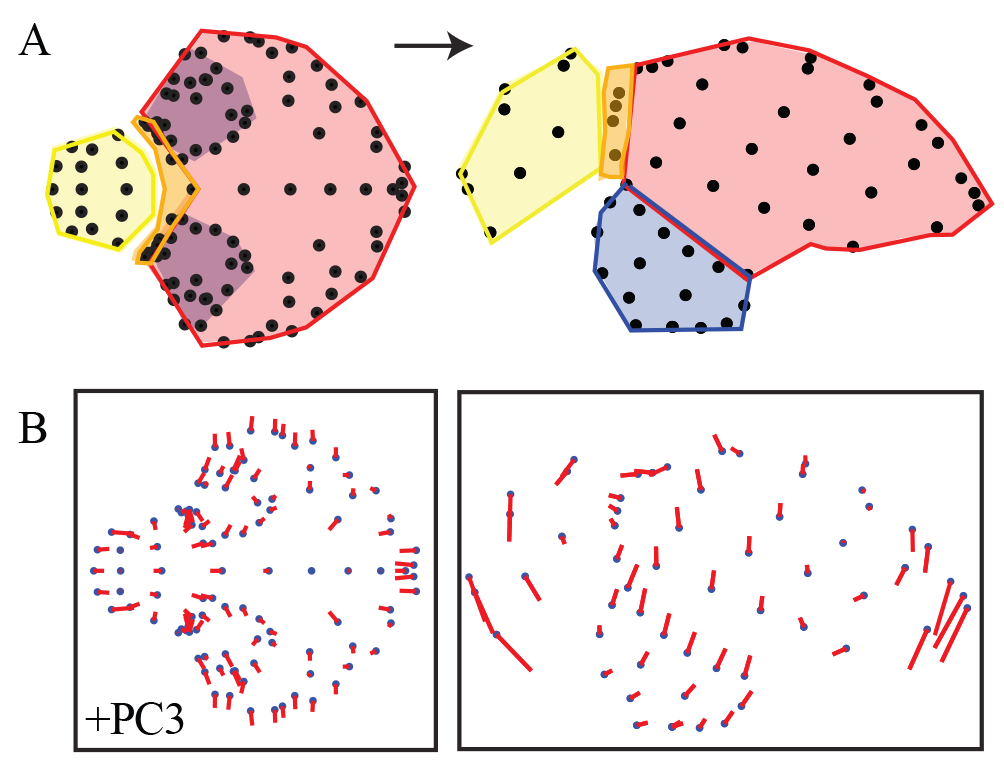


Figure S5: Shape changes along PC3 indicate shape at the positive end of PC3 for B) the Aves and Coelurosaur datasets in dorsal (left) and right lateral (right) views. A) Color-coded key of endocasts regions: Red - cerebrum, orange – intersection of cerebrum and cerebellum, yellow – cerebellum, blue – optic lobe. Dots represent mean landmark positions, and red lines in B and C represent the direction and at the maximum positive range of the specimens on PC3. Arrow indicates anterior.


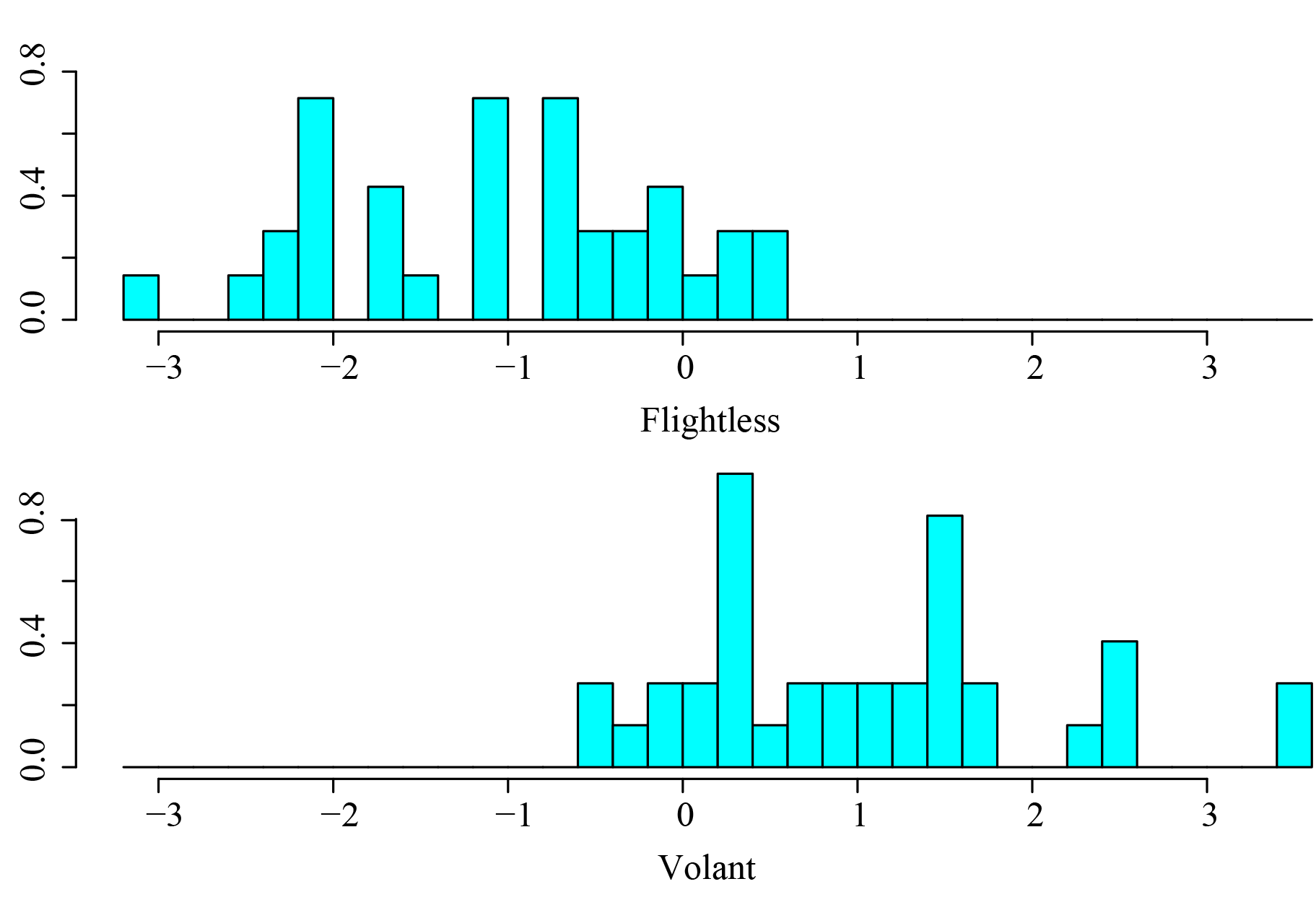


Figure S6: Bar plot of the single linear discriminant axis constructed from the LDA of endocranial shape variation in the Aves dataset. Top: flightless specimens; Bottom: volant specimens.
